# Age-Stratified Infection Probabilities Combined With a Quarantine-Modified Model for COVID-19 Needs Assessments: Model Development Study

**DOI:** 10.2196/19544

**Published:** 2021-05-31

**Authors:** Vena Pearl Bongolan, Jose Marie Antonio Minoza, Romulo de Castro, Jesus Emmanuel Sevilleja

**Affiliations:** 1 Department of Computer Science University of the Philippines Diliman Quezon City Philippines; 2 Center for Informatics University of San Agustin Iloilo Philippines; 3 National Center for Mental Health Mandaluyong Philippines

**Keywords:** COVID-19, epidemic modeling, age stratification theory, infection probability, SEIR, mathematical modelling

## Abstract

**Background:**

Classic compartmental models such as the susceptible-exposed-infectious-removed (SEIR) model all have the weakness of assuming a homogenous population, where everyone has an equal chance of getting infected and dying. Since it was identified in Hubei, China, in December 2019, COVID-19 has rapidly spread around the world and been declared a pandemic. Based on data from Hubei, infection and death distributions vary with age. To control the spread of the disease, various preventive and control measures such as community quarantine and social distancing have been widely used.

**Objective:**

Our aim is to develop a model where age is a factor, considering the study area’s age stratification. Additionally, we want to account for the effects of quarantine on the SEIR model.

**Methods:**

We use the age-stratified COVID-19 infection and death distributions from Hubei, China (more than 44,672 infections as of February 11, 2020) as an estimate or proxy for a study area’s infection and mortality probabilities for each age group. We then apply these probabilities to the actual age-stratified population of Quezon City, Philippines, to predict infectious individuals and deaths at peak. Testing with different countries shows the predicted number of infectious individuals skewing with the country’s median age and age stratification, as expected. We added a Q parameter to the SEIR model to include the effects of quarantine (Q-SEIR).

**Results:**

The projections from the age-stratified probabilities give much lower predicted incidences of infection than the Q-SEIR model. As expected, quarantine tends to delay the peaks for both the exposed and infectious groups, and to “flatten” the curve or lower the predicted values for each compartment. These two estimates were used as a range to inform the local government’s planning and response to the COVID-19 threat.

**Conclusions:**

Age stratification combined with a quarantine-modified model has good qualitative agreement with observations on infections and death rates. That younger populations will have lower death rates due to COVID-19 is a fair expectation for a disease where most fatalities are among older adults.

## Introduction

The initial impression that came out of Wuhan, China, in late 2019 and early 2020 was that COVID-19 most affects older adult males with pre-existing conditions. Classic compartmental models like the susceptible-exposed-infectious-removed (SEIR) model all assume a homogenous population, and that everyone has equal chances of getting infected. The SEIR model is initialized by “dividing” the population into four compartments; people “progress” through being susceptible, to getting exposed, to being infectious, to getting removed, either via recovery with permanent immunity or death. Permanent immunity is a common assumption when modelling viral infections, and it is assumed here. In future, the model may be modified for temporary immunity, as soon as we get reliable data on reinfection rates. A scan of the preprints from various modeling efforts during the first quarters of 2020 gave high estimates for the peaks of the exposed and infectious groups (40% of the population, by some estimates). Even our quarantine-modified model suffered from this, and this inspired us to use age-stratified infection probabilities, which gave us a lower bound for estimates.

## Methods

### Estimates by Age Stratification

**Figure 1:**
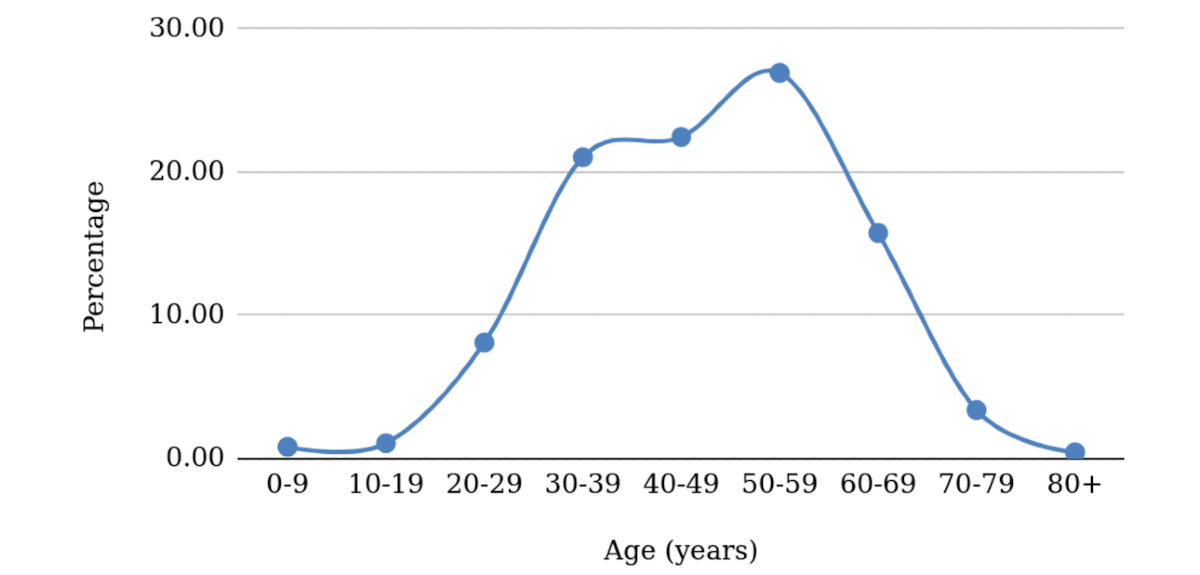
Projected age-stratified percentages of cases for China.

This calculates the “mathematical expectation” of future infections per age group, by multiplying an age group’s infection probability by the population in that age group. Initially, we used the data of patients with COVID-19 in Hubei [1], stratified by ages. As data came in, we repeated the calculations with updated Quezon City data. We treat the percentages of incidence in each age group as a proxy or estimate for the corresponding probabilities of infection for people in the corresponding age group. The true probabilities are and continue to be unknown, but the scatter of the data from Hubei is consistent with the virus affecting older adults with pre-existing conditions more than other groups (Figure 1).

**Figure 2:**
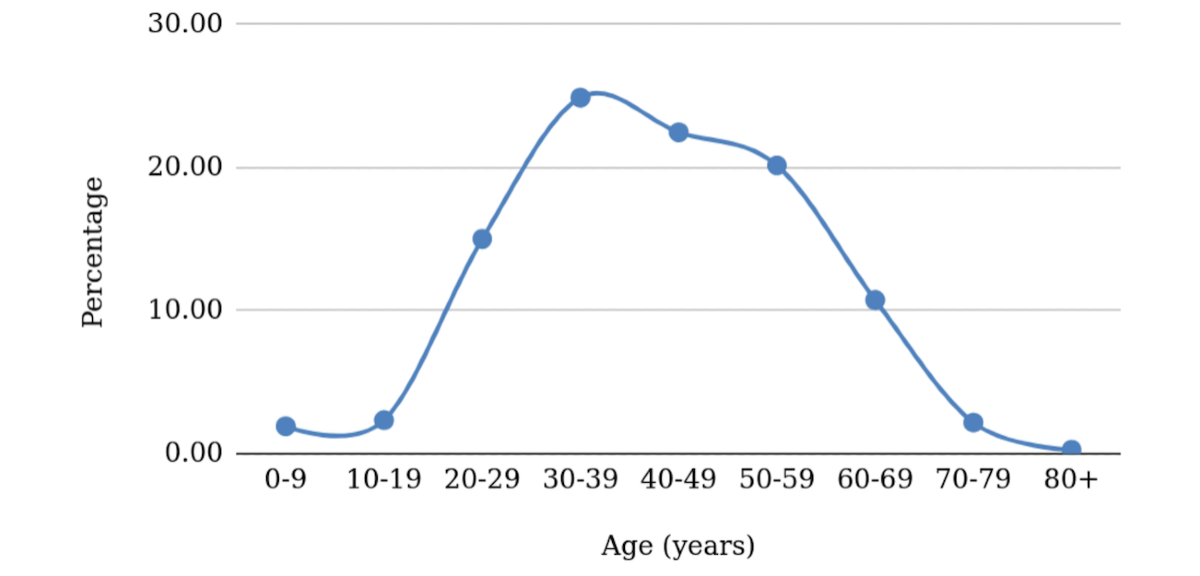
Projected age-stratified percentages of cases for the Philippines.

Next, we took the proxy probabilities from Hubei, and applied them to the actual age stratification of the Philippines. Due to its young population, this resulted in a “skewed to the right” distribution (Figure 2) compared to the Hubei distribution, and the true distribution for the study area will be revealed as actual cases are reported.

The Philippines has a median age of 25.7 years [2] compared to China’s 38.4 years [2]. This means half of the population is aged <25.7 years, so more than half of the population will be in the “safer” age groups, with lower probabilities of getting infected, and significantly greater chances of survival if they should contract the disease. Those who do get infected account for only 10.2% of cases (see Table 1, sum of the percentages of those aged 0-29 years old). We expect this to be true for other countries with low median ages.

**Figure 3:**
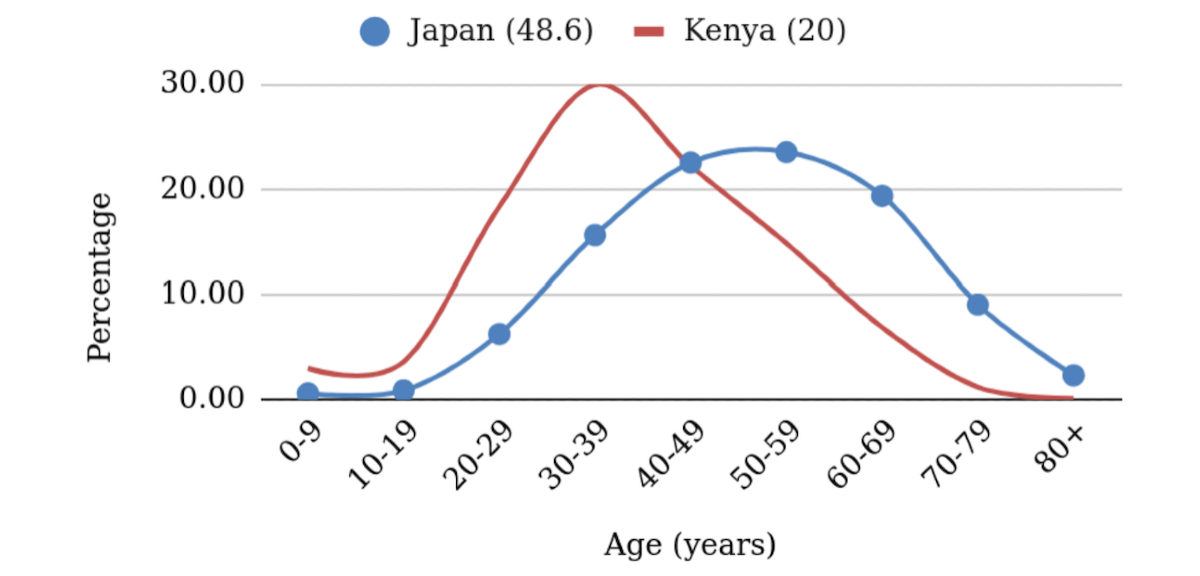
Projected age-stratified percentages of cases for Japan and Kenya. The average age in each country is given in parentheses.

Using the United Nations World Population Prospects 2019 data [3], we did a similar experiment with Japan (median age 48.6 years) and Kenya (median age 20 years) [2]. We see the younger population also skewing right (Figure 3). Martinez [4] did similar calculations, but did not treat the age-stratified incidence as a proxy for infection probability.

**Table 1:** Quezon City, Philippines, projection of COVID-19 cases and mortality using COVID-19 data from Hubei, China.

Age group (years)	Hubei, China		Quezon City, Philippines		
	COVID-19 cases, %	Case fatality rate, %	Projected COVID-19 cases, n	Projected COVID-19 case distribution, %	Projected mortality, n
0-9	0.9	0	5164.60	1.60	0.00
10-19	1.2	0.2	7345.98	2.28	14.69
20-29	8.1	0.2	53,444.14	16.57	106.89
30-39	17	0.2	87,156.90	27.02	174.31
40-49	19.2	0.4	75,013.55	23.25	300.05
50-59	22.4	1.3	61,753.09	19.14	802.79
60-69	19.2	3.6	27,420.07	8.50	987.12
70-79	8.8	8.0	4588.82	1.42	367.11
≥80	3.2	14.8	699.83	0.22	103.57
Total	100	28.7	322,586.99	100	2856.54

### Estimates Using a Quarantine-Modified SEIR Model

The quarantine-modified SEIR equations are shown below.

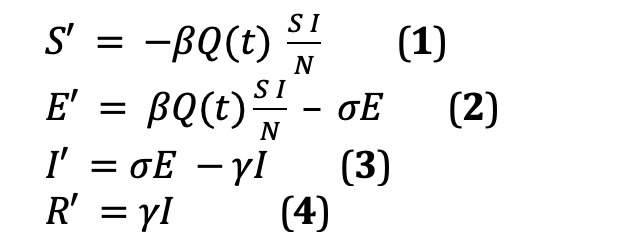



The insertion of the *Q(t)* term serves to control the *S × I* or susceptible-infectious interactions, hence lowering exposure. *Q(t)* equal to one means no quarantine (ie, no change to the model). A *Q(t)* value of 0.4 means a 60% effective quarantine, while *Q(t)*=0.6 means the quarantine is only 40% effective; therefore, the lower the *Q(t)*, the better. We allowed *Q(t)* to vary day by day (since cases began before the quarantine), and we also estimated the success of the quarantine. Henceforth, we refer to this model as Q-SEIR. Solution was via the Euler method, and time stepping was one day. We used Excel (Microsoft Corp) worksheets for our calculations.

The model was ground-truthed to the estimated number of exposed individuals at the national level on April 1, 2020 (N=7400) [5]. From the nationally reported number of exposed individuals (patients under investigation + persons under monitoring), Quezon City represents almost 10% of cases (~740); the Q-SEIR model predicted 705 cases.

## Results

Applying the Hubei infection probabilities on Quezon City with an age distribution as shown in Table 1 (from the 2015 Census, projected to 2020 at a 2% growth rate [6]) gave an estimate of 322,586 infectious individuals (accumulated, which we equate with the Q-SEIR peak), which accounts for less than 10% of the population of Quezon City. Deaths were predicted at 22,390 cases (6.94%), which lies between the World Health Organization (WHO) morbidity estimates of 5.58% [7] and the 12.47% reported in Italy [8]. These estimates were what was available as of April 2020, and will be updated in the discussion.

## Discussion

### Principal Results

Initial reports (around April 2020) estimated the Philippines’ death or mortality rate to be at 4.70% (4.05%-5.43%) [8]. This high estimate may be explained by sampling bias, wherein severe cases may have been overrepresented because of a lack of testing. Those who are infectious but are asymptomatic or exhibit mild symptoms should also be equally represented in the testing guidelines (at the moment, they are not), as well as those who were infectious with no symptoms and have recovered.

We tried the calculations from [4] using the death rate, which was reported at 2.3% for China [1]. This gave a much lower number of around 2857 deaths, for a Quezon City death rate of 0.89%. This figure is surprisingly low compared to the 6.94% projected using the estimated infection probability. The latest estimate of the Philippine COVID-19 death rate is 1.82% [9].

The delay in test reporting (estimate of 5-7 days [10]) factors in the estimation of the initial E-I-R values. In addition, this delay is compounded by the incubation period and, in our opinion, moves the quarantine effect further down from the actual date of implementation (March 15, 2020). We started the Q-SEIR simulation on March 20, 2020, with no quarantine assumed because the steep jump in cases occurred on this date; 60% effective quarantine was set for April 2, 2020.

The Q-SEIR model predicted 14.00% of the population will be infectious (I) at the peak. The two methods now give us a low and high estimate for Quezon City: infectious individuals will peak between 9.95% (from age stratification) and 14.00% (from Q-SEIR) of the population, around the third week of May 2020. These figures seemed high compared to the reported incidence during the same period, but were not in any way unique (compared to modeling done in other countries). At that time, the suspicion was that actual cases were undertested/underreported by as much as 90% (ie, only 10% of cases were being detected). Nevertheless, this range of values serves as a guide for planners in anticipating the need for personal protective equipment, mass testing, hospital beds, and other basic needs.

**Figure 4:**
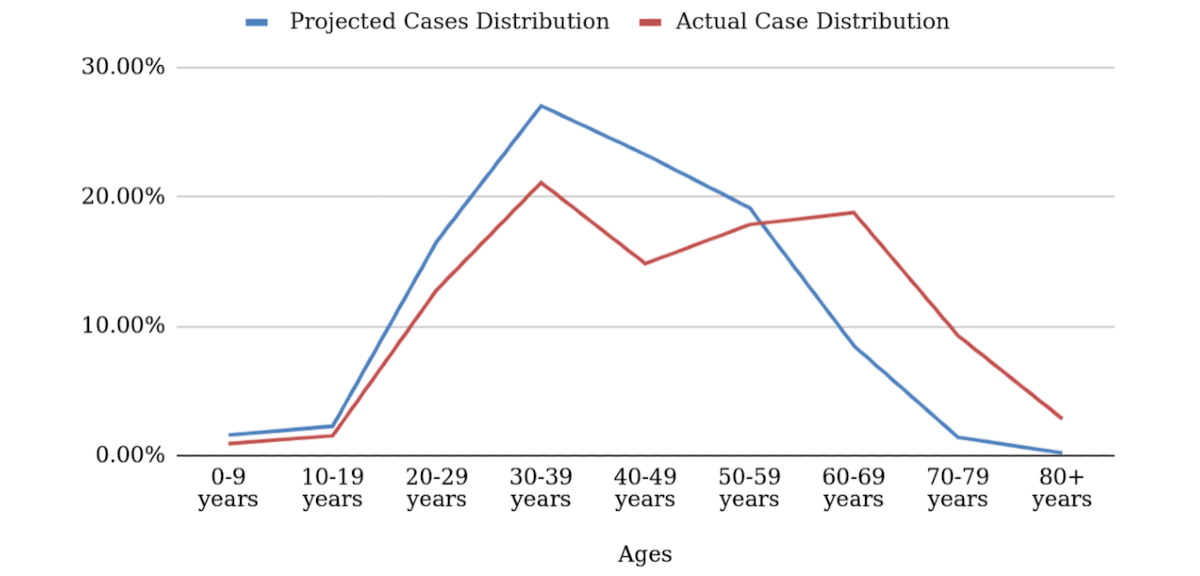
Projected versus actual age-stratified case distribution for Quezon City.

Figure 4 shows the scatter of cases for Quezon City projected by the Hubei data, and the actual scatter as of May 2020.

### Limitations

Like many research groups, we were and continue to be hampered by a lack of reliable data in a usable format. Even now, we refer to multiple data sets including the Philippine Department of Health’s DataDrop, WHO, and other data sources.

### Comparison With Prior Work

We were one of the first groups to forward the theory of age stratification when it comes to modeling COVID-19 infections. Recently, we came across works by Balabdaoui et al [11], Undurraga et al [12], and the WHO [13]. However, we are uniquely using incidence percentages as proxies for infection probabilities with good results.

### Conclusions

In conclusion, age stratification predicted the scatter of cases for Quezon City fairly well. It also predicted later observations of lower cumulative confirmed cases for the same time period (eg, 6587 cases per million compared to Italy’s 58,417 cases per million as of March 28, 2021); there is a color-coded world map in [14]. Some African countries have lower median ages than the Philippines, and they have generally lighter colors than Europe and North America. The best prediction is the noticeably lower death or mortality rate of 1.82% compared to Italy’s 3.05% and Indonesia’s 2.7% (all as of March 29, 2021) [9]. Since COVID-19 disproportionately affects older adults more than younger populations, we expect the Philippines to have a lower mortality rate than countries with older populations (eg, Italy has a median age of 47.3 years and Indonesia has a median age of 29.7 years, compared to 25.7 years in the Philippines). Later work can use actual infection probabilities to include the effect of age stratification in the model.

## Abbreviations

SEIR: susceptible-exposed-infectious-removed
WHO: World Health Organization
